# Challenges of functional imaging research of pain in children

**DOI:** 10.1186/1744-8069-5-30

**Published:** 2009-06-16

**Authors:** Simona Sava, Alyssa A Lebel, David S Leslie, Athena Drosos, Charles Berde, Lino Becerra, David Borsook

**Affiliations:** 1 P.A.I.N. Group, Department of Radiology, Children's Hospital Boston, Massachuesetts, USA; 2 P.A.I.N. group, Department of Anesthesiology, Children's Hospital Boston, Massachuesetts, USA; 3 Department of Anesthesiology, Children's Hospital Boston, Massachuesetts, USA; 4 Department of Medicine, Children's Hospital Boston, Massachuesetts, USA; 5 Department of Psychiatry, McLean Hospital Belmont, Massachuesetts, USA

## Abstract

Functional imaging has revolutionized the neurosciences. In the pain field it has dramatically altered our understanding of how the brain undergoes significant functional, anatomical and chemical changes in patients with chronic pain. However, most studies have been performed in adults. Because functional imaging is non-invasive and can be performed in awake individuals, applications in children have become more prevalent, but only recently in the pain field. Measures of changes in the brains of children have important implications in understanding neural plasticity in response to acute and chronic pain in the developing brain. Such findings may have implications for treatments in children affected by chronic pain and provide novel insights into chronic pain syndromes in adults. In this review we summarize this potential and discuss specific concerns related to the imaging of pain in children.

## Introduction

### Chronic pain

The International Association for the Study of Pain (IASP) defines chronic pain as "*an unpleasant sensory and emotional experience associated with actual or potential tissue damage, or described in terms of such damage*" [1]. Pain is considered chronic after 6 months of onset. However, the majority of pain studies set the minimum time at 3 months for pain to be considered chronic. Nevertheless, pain can be considered chronic when it does not resolve in the expected time frame after an acute injury, and does not respond to analgesic treatments. Chronic pain is a common and persistent problem in adult populations worldwide [2,3]. A recent multi-national survey study (N = 42,249) reported a prevalence of 37.3% – 41.1% for chronic pain conditions. Chronic pain of moderate and severe intensity can have serious deleterious effects on the mental health, employment status, sleep and personal relationships of affected individuals [3,4].

Our understanding of the effect of chronic pain on cortical, subcortical and brainstem neural networks has been greatly advanced with the introduction of non-invasive neuroimaging techniques. Pain is a subjective experience that is not only or necessarily determined by the intensity of the noxious stimulus [5], but also by a variety of biological and psychosocial factors, such as sex hormones, emotions, memories, or social expectations. It is therefore not surprising that the associated structural and functional changes are widespread and can be observed in brain areas not directly implicated in 'classic' pain processing. Neuroimaging findings have fundamentally altered the way in which we should evaluate and probably treat pain: as a disease rather than a symptom [6] and a disease predominantly affecting the brain [7].

Brain imaging studies of chronic pain in pediatric populations offer unique opportunities to understand changes in the young brain. Aside from providing novel insights into CNS processing of pain in children, these studies allow investigation of the disease from both a developmental and neuroplastic perspective. In the pediatric population, the brain is undergoing rapid changes, is more plastic, and may have an increased ability to recover after injury. Currently, the long-term effects of early pain on neural systems are not well understood, but findings of early traumatic experiences (e.g., surgery, immunization, etc) resulting in persistent changes in CNS have been reported [8],. Ideally, the use of non-invasive imaging methods to objectively evaluate changes in the brain in pediatric patients will lead to new treatment approaches that could potentially limit the development of long-term consequences.

### New insights into brain function in pain

Several non-invasive imaging techniques have been applied in research to investigate the brain areas involved in processing of acute and chronic pain, as well as the long term structural and functional changes occurring in the brain of chronic pain sufferers. Most functional imaging studies have been performed in adult volunteers or patients. Despite the heterogeneity of the clinical pain syndromes, a "central pain matrix" composed of primary nociceptive areas commonly activated by painful stimuli has been described [9]. Furthermore, additional brain areas are involved in processing the emotional [10] and cognitive [11] aspects of the pain experience, and their activation is dependent on the particular set of circumstances for each individual [6].

Imaging studies in adults have also helped uncover the central modulation of pain systems. By using distraction during painful stimulation, for example, Bantick and colleagues [12] showed that many areas involved in pain processing displayed reduced activation, supporting the behavioral observations of reduced pain perception. Studies of the placebo effect also provide evidence that the experience of pain can be altered by cortical mechanisms [13], suggesting that the sensory experience can be shaped by one's attitudes and beliefs [13]. The anterior cingulate and frontal cortices are part of a descending pain modulatory system that exerts top-down influences on the periaqueductal grey (PAG) and posterior thalamus to gate pain modulation [14]. Other regions, including the nucleus cuneiformis (NCF), have also been shown to be involved in modulation of pain in human imaging studies [6,15]. If an individual can learn to control the activation of these cortical areas through biofeedback, this might provide a different approach to treating disease. Biofeedback using real-time magnetic resonance imaging has been successfully applied in a group of chronic pain patients [16]. Subjects successfully learned to control the activation of the anterior cingulate cortex, and this process led to significant reductions in the magnitude of experienced chronic pain [16]. Finally, brain networks activated by empathetic pain (observing pain in a close friend or loved one) are similar to those activated by pain resulting from somatic inputs in the same individual [17].

Imaging research has also contributed to our understanding of the changes that occur in the brain of adult chronic pain sufferers. The brain in chronic pain is not simply processing heightened pain information; rather, neuronal networks of pain-transmitting areas undergo plasticity that results in long-term functional and structural reorganization, and ultimately influence the sensory, affective, and cognitive perceptions related to pain [6,18]. The role of the brain mechanisms in maintenance of chronic pain is apparent in some conditions such as chronic regional pain syndrome or fibromyalgia, in which the pain appears to result from abnormalities of central pain processing leading to hyperalgesia (i.e., increased response to normal painful stimuli) and allodynia (i.e., pain in response to normally non-painful stimuli), rather than from damage of peripheral structures [19]. In addition, structural imaging studies have shown that significant atrophy is associated with chronic pain [20], raising the possibility that chronic pain could also be considered a degenerative disease.

Together, these studies inform our approach to therapy. Most drugs that are currently used in the treatment of chronic pain (opioids, antidepressants and anticonvulsivants) are not able to control the pain in most patients, and in controlled clinical trials they have a ceiling effect of approximately 30% efficacy level [20] in pooled patient sets. In this context, neuroprotective drugs or drugs targeting sensory and emotional brain circuits might prove beneficial. Brain imaging techniques may be useful in monitoring the disease process and progress and the responses to specific analgesia and experimental pain [7].

### The impetus to evaluate changes in CNS function in children

Compared to the wealth of data from adult studies, the research investigating brain changes in children with chronic pain is still in its infancy. While numerous studies have documented the increased prevalence in children of chronic pain conditions, such as headache, abdominal, limb and back pain [21-23], as well as the long-term physical, psychological and social consequences of childhood pain (see below), very few studies have addressed the question of brain changes in pediatric pain populations. These studies are necessary in order to understand the effects of pain on brain maturation and plasticity processes. Indeed, many early experiences resulting in psychosocial or physical trauma in early childhood may eventually unfold in the form of generalized pain symptoms similar to those observed in depressed patients [24], patients with fibromyalgia [25] or in patients with post-traumatic stress disorder [26].

## Chronic pain in children

### Pain in children – prevalence

Prevalence rates of chronic pain in children reported in the literature are variable, depending on the definition, method of reporting and type of pain, as well as the characteristics of the study sample (age, gender, age of onset and duration of illness). For example, McGrath and colleagues [27] investigated chronic pain (defined as pain present for more than 3 months) in children with enuresis, cancer, and arthritis and found that the prevalence of chronic pain was 2.2% for enuresis, 12.5% for cancer, and 78% for arthritis. One epidemiologic study that investigated the prevalence of chronic pain regardless of etiology found that 25% of the 5336 children aged 4–18 years included in the study were affected by chronic pain [23]. A survey that investigated four of the most frequent pains (headache, stomach, back and limb pain) in a sample of 2465 adolescents aged 12–15 years revealed that 16.5% complained of pain that occurred at least weekly; moreover, 6.5% of subjects reported having pain in more than one location [22]. Van Dijk and colleagues [28] surveyed 495 schoolchildren aged 9 to 13 years and found that 57% of children experienced at least one recurrent pain (headache, stomach pain, muscle pain or growing pain). Six percent of the subjects were identified as having a history of chronic pain or having chronic pain at the time of the study [28]. An even higher prevalence of chronic pain, 30.8% (defined as pain present for more than 6 months) has been reported in a survey of German children and adolescents aged 4–18 years [21]. Despite treatment, a considerable proportion of children and adolescents continue to experience long-term pain. In a study of 254 children and adolescents aged 0–18 years with chronic pain, it was found that 48% of the subjects continued to experience pain one year after the original assessment, and 30% continued to have pain at a two year follow-up [29]. There are several methodological factors that influence the outcome of prevalence studies in chronic pediatric pain, related to the definition of recurrent and chronic pain, sampling techniques and methods of data collection. Despite the variability in the reported prevalence rates, these studies indicate that chronic pain is a common complaint in childhood and adolescence.

### Chronic pain and behavior in children – long term consequences

Because chronic pain frequently results in higher use of medical services and medication, it is not only an individual patient concern, but also a public health concern. Chronic pain has a negative impact on the quality of life, performance and mood of the affected children, and adolescents and can cause social, emotional and financial consequences for the family [30]. The severity of the pain-associated problems experienced by children and their families varies considerably depending on the clinical population studied. The effect of pain on psychological well being of children and adolescents can be substantial and a considerable number of studies have shown that symptoms of depression, anxiety (general and pain-specific) and stress are common complaints in children suffering from recurrent childhood pain of various etiologies, particularly when the pain is severe and causes disability [30,31]. The prevalence of depression in 13–18 year old adolescents that experience daily pain was three times higher (45%) than in the general population (16%) [32,33]. In addition to the pain intensity, depression can also be a predictor of functional disability [31] and interdisciplinary cognitive-behavioral treatment focusing on disability. Children and adolescents that experience pain are also at increased risk of missing school [32], and some have adjustment problems related to peer rejection and isolation [34]. These problems have been linked to academic underachievement, involvement with antisocial peers and unemployment [35].

In addition to problems encountered during childhood, chronic pain predisposes an individual to somatic and psychosocial consequences that extend into adulthood, specifically, increased reports of pain, disability and psychiatric symptoms [36,37]. Infants who have major surgery in the first 3 months of life show greater pain responses and require more intra-operative pain management during subsequent operations [38]. Even less noxious stimuli, such as heel prick, can result in increased sensitivity to mechanical stimulation lasting at least during the first year of life [39], suggesting that abnormal plasticity occurs in the pain pathways in the sensory connections in the dorsal horn, but possibly at higher levels in the spinal cord and even the brain. Several long-term follow-up studies of subjects that experienced recurrent pain during childhood found that they continue to experience pain during adulthood. For example, about 60% of children who experienced migraine headaches during childhood and early adolescence were still experiencing migraines 23 years later [40], and a relatively large percentage of adult daily headache sufferers report the initial onset of symptoms early in life [41]. The relative higher impact on long-term functioning of chronic pain in childhood compared to adulthood is not surprising. The nervous system is more plastic during childhood to allow for developmental and maturational processes to follow their course. Abnormal stimuli such as recurrent pain may affect plasticity in the peripheral and central nervous systems and may lead to long-term pain-related effects influencing a wide range of functions, including nociceptive processing, emotional processing and coping behaviors [42]. Pediatric patients that survive severe injuries have high incidence of post-traumatic stress disorder and depressive symptoms in the days, weeks and months following hospitalization, and these symptoms are associated with long-term functional impairment and diminished quality of life [43]. Therefore, early therapeutic interventions are essential in learning adaptive rather than maladaptive coping strategies and prevention of long-term negative outcomes of childhood pain.

## Functional imaging – opportunities for Advances

### Imaging techniques – insights into functional, chemical and anatomical changes in the brain

**Figure 1:**
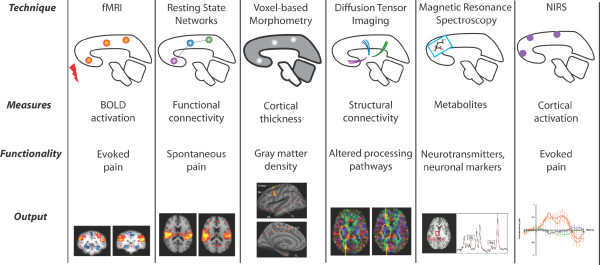
**Imaging Methods used in Pain Research**. See text for details.

A schematic of the imaging techniques used in pain research is presented in Figure 1. Below we provide detailed descriptions of these techniques.

#### Functional

Functional imaging techniques have revolutionized the field of neuroscience research. Functional magnetic resonance imaging (fMRI) is a non-invasive technique that assesses cortical activation by measuring changes in the local concentration of paramagnetic deoxyhemoglobin. This method has been referred to as **blood oxygen level-dependent **(BOLD) imaging. In a BOLD experiment, regional neuronal activation is associated with changes in blood flow and blood volume, generally leading to a washout of deoxyhemoglobin, which results in an increase in local signal intensity [44]. Functional MRI defines dynamic changes in blood flow with relatively high spatial resolution, and is a powerful tool that can be used to investigate neuronal networks involved in cognitive processing and the effects of disease states on brain functioning. Because it is non-invasive, fMRI can be used repeatedly in children, therefore allowing longitudinal studies of the development of neural networks during childhood and adolescence, evolution of disease processes and treatment effects.

Recent fMRI studies investigating the BOLD signal in adults have found that several brain areas show higher activation during periods of quiet rest compared to intervals when participants engage in attention demanding cognitive tasks. These brain areas have strong functional and anatomical connections and they form a "**resting state network**" (RSN) that is consistently found across subjects [45]. Recent studies have shown that the activity of the "default network" is disrupted in several pathological conditions, including chronic back pain [46] and depression [47]. Measures of the RSNs provide insight into the functional brain connectivity during a resting state and have the potential to measure therapeutic efficacy [7].

**Cerebral near-infrared spectroscopy **(NIRS) is a non-invasive technique that can detect subtle changes in the concentration of natural chromophores such as oxygenated and de-oxygenated hemoglobin. NIRS has been successfully applied in newborns, children and adults to measure the hemodynamic and oxygenation changes related to cortical processing of specific stimuli. In the field of pain research, NIRS studies have documented that painful stimuli elicit specific hemodynamic responses in the somatosensory cortex, implying conscious sensory perception in preterm neonates [48]. NIRS seems to have a great potential in pain measures; for example, a recent paper has indicated a specific signal for pain [49] similar to that observed in previous fMRI studies [10].

#### Chemical (MRS)

Magnetic resonance spectroscopy (MRS) provides an excellent tool to study alterations in neurotransmitters and neuronal markers in the brain in vivo. Different types of MRS techniques have been developed, each providing unique information about the brain chemistry. For example, proton spectroscopy (^1^H-MRS) allows measurement of glutamate, glutamine and gamma-aminobutyric acid (GABA), as well as N-acetyl aspartate (NAA), a neuronal marker involved in synaptic processes [50]. Phosphorus spectroscopy (^31^P-MRS) can detect phosphorus-containing compounds such as phosphodiesters, phosphomonoesters and phosphocreatine, which are markers of membrane integrity and energy use in brain cells [51]. Fluoride spectroscopy (^19^F-MRS) allows for measurements of fluorinated drug pharmacokinetics [52]. The MRS approach has been applied in several pain conditions including migraine [53] back pain [54] and spinal cord injury [55] and it has great potential of providing biomarkers of disease that precede structural changes in the brain [7].

#### Anatomical

**Diffusion Tensor Imaging (DTI) **is a MRI technique that measures changes in white matter tracts [56] based on microstructural changes in water diffusion. Using this approach, functional anisotropic differences in normal vs. abnormal tracts can be inferred based on DTI measures. The technique has been used in a number of pain disorders such as migraine [57] and poststroke central pain [58] and may offer insights into the underlying changes in brain state. Although there are some limitations to DTI [59], when combined with fMRI studies, it may help improve our understanding of functional anatomical mapping of processing information.

Information about structural and functional organization of the brain can also be inferred from MRI data. Cortical thickness measurements reflect the size, density, and arrangement of neurons, glial cells and nerve fibers. Recent studies have shown that regions that are axonally connected have strongly correlated cortical thickness measurements, possibly reflecting the underlying cytoarchitecture and neural connectivity [60]. Therefore, analyses of the whole-brain cortical thickness data allow identification of large-scale anatomical networks, providing a different method to investigate the normal cerebral development and cortical abnormalities in various neuropsychiatric disorders, as well as validate the findings of functional networks studies [61].

### Imaging children – concerns and considerations

Magnetic resonance imaging is non-invasive and can be used repeatedly in children, therefore allowing longitudinal studies of the development of neural networks during childhood and adolescence, evolution of disease processes and treatment effects. Functional magnetic resonance imaging (fMRI) is a powerful tool that can be used to investigate neuronal networks involved in cognitive processing and the effects of disease states on brain functioning.

#### Ethical considerations

The Nuremberg Code and the Helsinki Declaration were among the first documents to establish principles of proper and responsible conduct of human experimentation in medical research. The Belmont Report (National Commission for the Protection of Human Subjects of Biomedical and Behavioral Research, 1978) provided basic ethical principles that should govern research involving human subjects and supported the protection of vulnerable populations. The three fundamental ethical principles outlined in the Belmont report are *respect*, *beneficence *and *justice*, and the interpretation of these principles is different in pediatric vs. adult populations. *Respect *refers to the recognition and protection of the autonomy of all individuals. Certain vulnerable populations have reduced autonomy because of young age, illness, mental disability, or situations that restrict their liberty and have impaired ability to provide free informed consent. In these situations, the appropriate level of protection is a matter of balancing the principle of respect for persons with the need to protect vulnerable populations [62]. In addition to obtaining consent from the parents, children who have the intellectual maturity should be given the opportunity to assent (or dissent) to participating in research.

The *beneficence *principle refers to maximizing the benefits obtained from research while minimizing risks to the research subjects. Under US regulations, Institutional Review Boards (IRBs) can approve pediatric research that falls within one of 3 categories: (1) minimal risk, (2) more than minimal risk with the prospect of direct benefit and (3) minor increase over minimal risk and no direct benefit, but likely to generate important scientific knowledge [63]. To be ethically acceptable, the risk/benefit profile should be at least as favorable to the subject as the available alternative, including not participating in research [63]. Related to the risk of participating in research, the use of placebo trials in children has been controversial, because doing so might unnecessarily expose children to undue risk of physical or psychological pain and discomfort [64]. Instead, using an alternate therapy has been considered an acceptable solution. The benefits of research are broader than the individual direct benefit from a drug or procedure being investigated. Benefits include additional medical attention, and any physical or psychological tests a subject might receive as part of the research protocol. In addition, other children will benefit from medical advances that result from research. These benefits, as well as the risks associated with not doing research in children should all be considered when evaluating the risk/benefit profile of research studies [64]. The *justice *principle refers to the fairness of subject selection and equal treatment of all subjects. Because research carries both benefits and burdens, justice requires that no one socio-economic group receive disproportionate benefits or bear disproportionate burdens related to research. The NIH now requires that children must be included in all research studies supported by NIH, unless there are scientific and ethical reasons not to include them.

In the area of pediatric pharmacotherapy, *protection *meant excluding children from research. As a result, only about 20% of the drugs prescribed for children have been systematically tested for their safety and efficacy in pediatric populations [64]. Recently, legislative changes have led to an increased number of studies conducted in children. The Food and Drug Administration (FDA) can require companies submitting a new drug application to test the drug in children if pediatric use is anticipated, and the National Institute of Health (NIH) can offer contracts to fund studies in pediatric populations. Rather than restricting pediatric research because of the inherent challenges, it is important to allow children to participate in well-designed research studies so that they can benefit from research advances to the same degree as adult populations.

#### Growing brain – maturation of systems/processes

Human brain development is a non-linear process in which structural and functional maturation continue into early adulthood [65]. The brain areas associated with basic functions mature early (i.e., motor and sensory cortices and parietal areas involved in spatial orientation, speech and attention), while the frontal areas involved in higher functions (i.e., executive processing, attention, motor coordination) mature more slowly. Brain growth occurs most robustly during the first 3 years of life and brain weight reaches adult values (about 1.45 kg) between 10 and 12 years of age [66]. The increase in brain volume during the first three years of life reflects an increase in gray matter (i.e., development of dendritic trees and synaptogenesis) as well as fiber tract myelination [67,68].

The ratio of gray to white matter volume changes with age: gray matter increases until about age 8 and then decreases, while white matter increases until about age 30 before starting to decrease [69,70]. At the neuronal level, dendritic development and synaptogenesis occur in subcortical and cortical structures at different rates and at different ages. For example, synaptogenesis starts between 3 and 4 months of age in the visual and prefrontal cortices, but while the process is rapid and maximum synaptic density is reached between 4 and 12 months in the visual cortex, the development of synapses is slower in the prefrontal areas, where maximum synaptic density is reached around 1–2 years postnatally [68]. Significant reductions in the number of neurons through the process of programmed cell death (e.g., apoptosis) and pruning of synapses also occur during development in many brain areas, including visual cortex, medial amygdala, nucleus accumbens and the hypothalamus [71,72]. In the prefrontal cortex synaptic density decreases between two and six years of age and reaches adult levels by sixteen years of age [72].

The pattern of age-related neuroanatomical changes is paralleled by physiological changes. Cerebral metabolic rates are closely linked to the synaptic activity of cortical neurons. The overall resting activity of the gray matter regions as measured by glucose utilization using positron emission tomography (PET) is low at birth, increases after the first year of life and reaches a peak around four or five years of age [73]. At their peak, the metabolic levels are higher than adult levels, and are maintained until approximately nine years of age, subsequently decreasing to reach adult levels by the latter part of the second decade of life [73]. However, different cortical areas have different rates of functional maturation. The sensory-motor cortex, thalamus, brainstem, and cerebellar vermis are the first areas to show increases in glucose uptake, followed by the parietal, temporal, occipital and cerebellar cortices, and finally by the frontal and dorsolateral occipital cortices [65].

#### The plastic brain

Increased synaptic density in the developing brain presumably reflects the number of unspecified or labile synaptic contacts and provides the anatomical substrate for plasticity [68]. Critical periods are specific developmental windows when genetic and environmental processes interact to establish normal long-term functionality. Genetic or environmental insults occurring during these critical periods could lead to abnormal structural and functional rearrangements of the cerebral cortex. For example, increased alcohol consumption during adolescence can result in decreased volume of the prefrontal cortex and prefrontal cortex white matter [74]. From a therapeutic perspective, critical periods could allow for normalization of impaired functions. One striking example relates to the visual system: strabismic amblyopia can be reversed during childhood, provided that the good eye is occluded and the child is forced to use the squinting eye [68].

Plasticity is not limited to periods of brain development. The strength of synapses changes as a function of neuronal activity (i.e., activity-dependent plasticity) so that coherence of functional networks (or "cell assemblies") can either increase or decrease as a result of persistent synaptic activity. Activity-dependent plasticity in pain circuits has been proposed as a mechanism that may lead to a progressive increase in the response of the system to repeated stimuli [75]. In the peripheral nervous system (PNS), activity-dependent plasticity manifests itself through decreased threshold of the nociceptor terminals, and increased release of neuromodulators in the circuits of the dorsal horn [75]. The nociceptive pathways can exhibit reversible changes in the excitability of primary sensory and central neurons ("modulation"), as well as long-lasting alterations related to synthesis of neurotransmitters, expression of receptors and ion channels, or connectivity and survival of neurons in the network ("modification") [75]. These changes in the dynamics of neural networks could be related to pain behaviors, and could explain increased pain sensitivity to various stimuli (i.e., thermal and mechanical allodynia and hyperesthesia) in chronic pain sufferers [46]. Pain-induced plasticity can persist after the painful stimulus ceases and pain becomes a maladaptative process.

#### Morphing brains

Adult brains vary in shape and size between individuals. In order to analyze and interpret data from neuroimaging studies, researchers often perform spatial transformations on each subject's brain into a common anatomical frame of reference, either Talairach space (based on a single subject's brain [76] or, more recently, on population-based atlases such as the MNI305 atlas [77]. Non-linear spatial transformations have also been applied to imaging data, increasing the quality of inter-subject registration and allowing improved anatomical localization of BOLD activation [78,79].

In addition to the problems raised by performing transformations to a standard space, analysis of MR imaging data from children poses additional challenges. Firstly, child brains are more variable than adult brains and variability seems to be increased in children younger than six years of age [80]. Secondly, the size of the brain is smaller in children than in adults, but a child brain is not simply a reduced adult brain because complex maturational processes occur at different rates in different brains structures, as described earlier. Transforming pediatric brains into an adult-derived space by simple proportional downsizing of a grid system is likely to introduce additional bias into the analysis of fMRI data from young subjects. The bias is likely to be larger in data from children younger than 6 years, for smaller brain structures (such as the brainstem or subcortical regions), and for higher-resolution images [80,81]. To date, there are no pediatric brain atlases. In 1999, the National Institute of Neurological Disorders and Stroke (NINDS) initiated the Pediatric Neuroanatomic Study, a multicenter program that aims to establish a normative neuroimaging database for brain development in healthy children (0–18 years). It is expected that this study will provide data that can be used to generate age-specific brain atlases, which will greatly facilitate further advances of neuroimaging research in children. However, Burgund and colleagues [82] examined the variability of various brain regions in children aged 7–8 years (transformed into stereotactic space), and found that they differed only slightly from adults, thus validating the use of a standard, adult atlas in this pediatric population.

#### Blood-oxygen-level dependent (BOLD) signal

Similar to the inherent biases encountered when scaling pediatric brains to adult atlases, statistical thresholds used in pediatric research to identify signal changes are derived from adult studies and might not readily apply to data acquired from children. Identifying true activation in young subjects might be biased in the presence of differences in threshold of response, reactivity, or robustness of the response between pediatric and adult populations. For example, Thomason and colleagues [83] examined breath holding fMRI responses in children younger than 12 years of age and adults and found that the BOLD response is smaller and noisier in children than in adults, consequently producing less significant activation maps. During the early stages of development of different cortical areas, gray matter has a greater thickness and density in children compared to adults. For brain areas such as the prefrontal cortex that develop slowly, these differences are maintained until adolescence. Therefore, the magnitude of the signal acquired from gray matter areas should be corrected for volume averaging effect, and the correction should take into account the age of the subjects and the cortical area under investigation.

Several fMRI studies showed that children under the age of five have variable patterns of BOLD signal changes when compared to adults [84]. While adults show consistent positive BOLD responses in the occipital cortex in response to visual stimulation, children can have either positive or negative BOLD signal changes [85], suggesting that in young children the hemodynamic coupling may be different than in adults. However, after 8 years of age, the hemodynamic response functions are similar to the adult population [86]. The interpretation of these imaging studies is difficult because most of the young children were sedated, which has been shown to reverse the direction of the BOLD signal in adults [87]. In awake infants (aged three days to fourteen weeks old) investigated with near infrared spectroscopy, the increase in oxygen consumption in the visual cortex after visual stimulation outpaces the increase in blood flow, supporting the observation that the hemodynamic response in children is a reversal of the adult pattern [88].

Taken together, these studies underscore that special consideration has to be given to methodological factors when analyzing and interpreting fMRI data from infants and young children.

## Imaging pain

### Overview of pain imaging studies- adults

A recent meta-analysis of human studies of acute pain described a neural network composed of several areas that are consistently activated during pain perception. This network, sometimes termed the "pain matrix", included the thalamus, primary (S1) and secondary (S2) somatosensory cortices, insula, anterior cingulate cortex (ACC) and the prefrontal cortex (PFC) [89]. The activity of the pain matrix decreases during pharmacologically induced analgesia [90]. These areas perform parallel processing of the different aspects of pain. While thalamus, S1, S2 and parts of insula process the sensory-discriminative features of the painful stimulus (i.e., stimulus localization and intensity), the ACC and anterior insula process the affective-motivational aspects (emotion, arousal, selective attention) and the PFC responds to the cognitive aspects of pain (attention, memory, stimulus evaluation) [91,92].

The signature of chronic pain on the brain is partially distinct, and includes not only the pain matrix, but also brain regions critical for cognitive and emotional processing, such as the medial prefrontal cortex (mPFC), dorsolateral prefrontal cortex (DLPFC), parietal association cortex, amygdala, ventral striatum, and hippocampus [93,94]. Imaging studies have shown that reorganization occurs in several brain areas involved in sensory and affective processing of pain, such as the thalamus and the cortex. One study investigating patients with chronic back pain (lasting more than 6 months) showed regionally specific decreased gray matter volume in bilateral dorso-lateral prefrontal cortex (DLPFC) and right thalamus [95] suggesting that the pathophysiology of chronic pain includes thalamo-cortical processes. Both DLPFC and thalamus are involved in perception of pain, and DLPFC has been hypothesized to inhibit the orbitofrontal cortex (OFC), therefore decreasing the intensity of perceived pain [96]. The thalamus is an important relay in the nociceptive and sensory pathways from the spinal cord to the cortex and decreased thalamic gray matter may be related to the generalized sensory abnormalities associated with chronic pain [97]. One recent study [93] has shown that chronic spontaneous pain is associated with increased activation of the mPFC, a region involved in detection of unfavorable outcomes and processing negative emotions and response conflict [98]. Other studies also described reduced gray matter in brain areas related to pain sensation, memory, and associated emotional processing, such as the anterior cingulate cortex (ACC), anterior and posterior insula, orbito-frontal cortex, and parahippocampus [20,99,100]. Taken together, these studies support the idea that chronic pain may lead to structural changes in cortical and subcortical brain areas.

Further evidence for pain-related changes in the brain is provided by studies investigating brain chemistry. N-acetyl aspartate (NAA) is a neuronal marker involved in synaptic processes [50]. Decreased levels of NAA in the brain may reflect neuronal loss and degeneration, as well as long-term neurotransmitter changes. Grachev and colleagues [101,102] showed decreased levels of NAA in the DLPFC in patients with chronic low back pain or CRPS. Similarly, Sorensen and colleagues [103] found that patients with neuropathic diabetic pain have reduced NAA levels in the thalamus compared to diabetic patients without pain.

Results from functional studies using positron-emission tomography (PET) or fMRI suggest that brain function may be affected by chronic pain. Von-Frey stimulation of the affected limb in adult patients with CRPS evoked pinprick hyperalgesia and produced greater contralateral activation than identical stimulation of the unaffected limb in primary (S1) and secondary (S2) sensory cortex, insula, anterior cingulate cortex, and frontal cortices [104,105]. Mechanical allodynia evoked by brushing the affected limb was reported to correspond with activation of motor (M1) and cognitive regions (frontal regions), areas involved in emotional processing (e.g., anterior and posterior cingulate cortex, temporal lobe), parietal association cortices, as well as pain sensory regions (e.g., S1, insula) [104]. Of note was the significant negative activation in visual, posterior insular, and temporal cortices in response to brushing that evoked allodynia. In a recent study using magnetic source imaging, cortical reorganization was reported in the contralateral S1 cortex in patients with CRPS [105]. The reorganization involved parts of the body (lips and fingers) that did not have pain, but exchanged representations following recovery from CRPS. Functional cortical reorganization has also been described after limb amputation in primary somatosensory and motor cortices [106], and it has been related to phantom limb pain, rather than referred phantom sensations [18]. Cortical reorganization in patients with phantom limb pain also occurs in brain areas involved in processing affective-motivational aspects of pain, such as the insula, anterior cingulated cortex, and frontal cortices [107]. Taken collectively, these studies suggest that cortical plasticity in adults suffering from chronic pain is intrinsically maladaptive, particularly with respect to sensory-motor processing and that such changes are an essential feature underlying the pathophysiology of the disease.

During performance of cognitive tasks, the RSN shows functional reorganization and at least three canonical networks emerge. The "default-mode network" (DMN) is composed of brain regions that show decreases in activation and includes the ventromedial prefrontal cortex (VMPFC) and posterior cingulate cortex (PCC) [45,46,108]. Other emerging networks typically show increases in activation during cognitive tasks, including the central-executive network, which includes the DLPFC and posterior parietal cortex (PPC), and the salience network, which includes the ventrolateral prefrontal cortex (VLPFC), anterior insula, and the anterior cingulate cortex (ACC) [109,110]. Baliki and colleagues [46] showed that the functional connectivity within the DMN is altered in patients that had suffered from chronic back pain for an average of 6 years. Compared to healthy control subjects, patients with chronic back pain exhibited decreased deactivation in the mPFC, amygdala, and PCC during performance of an attention task. The extent of the mPFC deactivation was correlated with the number of years of pain suffering. In this study, performance on the attention task was similar between chronic pain and control subjects, suggesting that the differences in DMN connectivity were not related to the ability to complete the task. This study supports the idea that chronic pain has a widespread effect on brain function, affecting cortical circuits beyond those involved in perception. A second study investigated RSNs in female patients with complex regional pain syndrome and found increased connectivity between nodes of the salience network, including the bilateral insula and temporal pole, the bilateral cerebellum, and the left sensory-motor cortex; no changes were found in the vision-related network or the DMN between patients and healthy controls [111]. In this study, the pain severity was correlated with bilateral insular and temporal pole connectivity, whereas the duration of pain was correlated with dorsal anterior cingulate and hypothalamus/thalamus connectivity, suggesting that the brain changes are a consequence, rather than a cause of the increased nociceptive perception [111]. While these two studies report contradictory results in relation to the changes in DMN in chronic pain, it is important to note that brain reorganization is a plastic, time-dependent process that is initially driven by peripheral and spinal cord events, and subsequently by higher processing related to coping strategies [46]. Therefore, it is likely that the extent and the pattern of functional alteration in the DMN are related to the duration of chronic pain, as well as other pain characteristics (intensity and type of pain, presence of depression or anxiety).

In summary, findings from structural and functional imaging studies in humans suggest that the brain in chronic pain is not simply processing heightened pain information. The network of pain-transmitting areas within the central nervous system undergoes functional and structural reorganization in patients with chronic pain, and this central plasticity could in turn influence the sensory, affective, and cognitive perceptions related to pain.

### Pain imaging studies in children

#### Acute pain studies

Somatosensory-evoked potential studies have shown that from at least the 7th gestational month, the somatosensory pathways can conduct peripheral impulses to the cortex and the cortex is mature enough to produce responses [112]. As the pathways become myelinated during normal development, the latencies of the cortical responses decrease [113]. However, very little is known about central pain processing in infants and young children. Because the brain development and maturation continues after birth, and the affective and cognitive circuits are not fully developed in young children, it is likely that the pain experience has different dimensions in pediatric populations compared to adults. It is possible that the brain responses to the painful stimuli are also different in children.

To date, only two NIRS studies investigated brain changes during acute pain experiences in children. Slater and colleagues [114] measured changes in cerebral oxygenation over the somatosensory cortex in premature infants undergoing heel lances for routine blood sampling. The results showed that infants aged between 25 and 45 weeks gestational age exhibited clear cortical responses in the contralateral somatosensory cortex, and that the magnitude increased while the latency of the response decreased with age. A similar increase in the hemodynamic response in the somatosensory cortex has been described by Bartocci and colleagues [48] in preterm newborns (28–36 weeks of gestation) during venipuncture. In their study, somatosensory cortical activation was bilateral, and no increase in activation was observed in the parietal and occipital cortices, suggesting that preterm newborns might be consciously processing acute pain.

#### Chronic pain studies

To date, only one study has been published on brain changes in children with chronic pain. Using fMRI, Lebel and colleagues [115] investigated children nine to eighteen years of age with CRPS affecting the lower extremity. Unlike adult CRPS, the pain in pediatric CRPS frequently fluctuates and often resolves in less than 2 years, allowing comparisons of painful vs. pain-free states. Patients underwent two scanning sessions: the first one during an active period of pain, and the second one after symptomatic recovery. During active CRPS, patients experienced mechanical and thermal allodynia for the affected extremity, and BOLD activation patterns were similar to data reported in adults [104]. Activation changes were observed in pain-related areas (primary sensory-motor cortices, insula) and also in regions that presumably contribute to non-pain symptoms. These included the parietal, frontal and temporal cortices, which are thought to be involved in attention and other aspects of altered cognition, fear and anxiety [104,116]. Interestingly, the brain activation patterns continued to be different in response to mechanical and thermal stimulation of the affected vs. unaffected extremity, despite the absence of allodynia, suggesting that functional abnormalities in CNS circuitry may outlast the signs and symptoms of CRPS and could alter the pain processing later in life.

## Conclusion and future directions

The prevalence of chronic pain in children warrants further research to decipher the mechanisms of pain and potential therapeutic approaches. Research in children presents special challenges related to ethical treatment of children, technical adaptations necessary for acquiring and analyzing data, and interpretation of results from a developmental perspective.

Functional imaging of the changes that occur in the developing brain as a consequence of chronic pain experience is still an emerging field, and there is a considerable amount of information that we could learn from these studies. First, functional imaging studies during resting states or during peripheral stimulation of nociceptive pathways are essential in characterizing the brain networks that are modified by pain and the effects of affective and cognitive processing on these networks. Second, anatomical imaging studies can aid in uncovering the connectivity between nodes of the functional networks and provide insight into the cause of functional changes. Third, the investigation of chemical changes in the brain provides another approach for characterizing the CNS changes in chronic pain and may allow investigation of drug pharmacokinetics at target sites in the brain [52]. Because these techniques are non-invasive, they can be used repeatedly in longitudinal designs in order to assess the long-term changes in brain structure and function, and the effect of pain on normal development and maturation. Fourth, imaging methods could be useful in evaluating the response to therapy and could help development of new approaches in clinical trials.

The directions outlined above are essential in understanding the changes in neural systems produced by chronic pain, from an anatomical and functional level to human behavior and long-term effects on fundamental developmental and maturational processes.

## Competing interests

The authors declare that they have no competing interests.

## Authors' contributions

All authors have read and approved the final manuscript.

SS organized the conceptual frame and wrote most sections of the review. AAL contributed to writing the clinical and imaging sections. DSL contributed to writing the clinical sections. AMD contributed to writing the epidemiological and imaging sections. CB contributed to writing the ethical and clinical sections. LB contributed to the chronic pain and imaging sections. DB contributed to the conceptual framework and overall direction of the paper including reviewing and editing the paper.
